# *Piedraia hortae*: biofilm formation and its importance in the pathogenesis of *Piedra nigra* (black piedra)^☆^

**DOI:** 10.1016/j.abd.2023.12.005

**Published:** 2024-07-20

**Authors:** Hiram Larangeira de Almeida Junior, Thales Moura de Assis, Eduardo Camargo Faria, Luiz Roberto Kramer Costa, Berenice Marques Ibaldo

**Affiliations:** a Postgraduation in Health and Behavior, Universidade Católica de Pelotas; b Department of Dermatology, Universidade Federal de Pelotas, Pelotas, RS, Brazil; c Sector of Mycology, Laboratório Ary Costa, Pelotas, RS, Brazil

**Keywords:** Biofilms, Microscopy, Electron, Piedraia hortae

## Abstract

**Background:**

Little is known about the ultrastructure of *Piedraia hortae*.

**Objective:**

To examine a *P. hortae* colony with scanning electron microscopy and investigate possible contributions to its the pathogenesis of black piedra.

**Results:**

On low magnifications, two distinct aspects of the colony are identified, a compact area and a filamentous area. Analysis of the filamentous area demonstrates hyphae adhered by a thin reticular substance. A recurring finding is the adhesion between the fungal filaments in parallel. On high magnifications, the microfibrillar substance adhering the hyphae to each other becomes very evident. Examination of the compact area shows the hyphae embedded in the reticular matrix forming a biofilm and the colony well adhered. On high magnification, it can be observed that the hyphae are within this fibrillar matrix, which has the same appearance as the filamentous substance that adheres the hyphae to each other.

**Study limitations:**

Only one strain was examined.

**Conclusions:**

The formation of biofilm with fungal structures and reticulated extracellular substance is important in the pathogenesis of black piedra.

## Introduction

*Piedra nigra* is a well-known disease,^1^ which, together with *Piedra alba* (white piedra), constitutes a group of two similar diseases, also called trichomycosis^2^ or ectotrichomycosis,^3^ in which nodules appear on the hair shafts, dark or white respectively, without epidermal involvement.

*Piedra alba* is a condition resulting from the colonization of some species of the genus *Trichosporon*, such as *T. cutaneum*, *T. ovoides* and *T. inkin*, occurring on the hair shafts of the beard, armpits and pubis, with scalp hair less affected, varying from white to light brownish coloration. The genus *Trichosporon* includes filamentous fungi that can form complex biofilms.

Historically, *Piedra nigra* was described by Paulo Horta,^4^ who discussed in the original publication *Piedra nostras* from Europe and *Piedra colombica* from South America, both caused by non-pigmented fungi, also called nodular trichomycosis or trichosporia, as the genus *Trichosporum* had already been coined (in the spelling used at that time).

**Fig. 1:**
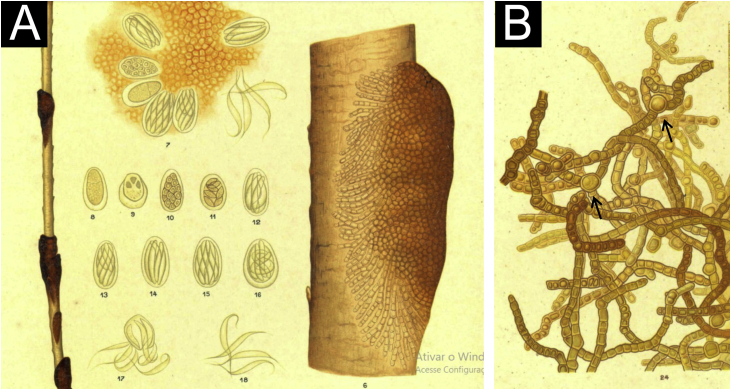
Drawings from the original 1911 publication. (A) Dark nodules on the hair shaft and the formation of ascospores (8 to 18). (B) Microscopic appearance of the etiological agent with hyphae, which show round dilations (arrows).

In that publication, he described cases of two young male students^4^ from Bahia different in clinical appearance, with dark nodules (Fig. 1A), as well as the fungal colonies obtained from them. Morphologically, the examination of the agent also showed differences, without the formation of yeast-like coccoid structures described for the *Piedra alba* agent. The hyphae, in addition to pigment, showed round dilations (Fig. 1B), chlamydospores, as well as ascospores (observed inside a saccular structure) at different evolution stages (Fig. 1A).

*Piedra nigra* is endemic to South America,^5^, ^6^, ^7^ and some indigenous populations show a prevalence of up to 50%, with cases also being described in Asia,^8^, ^9^ commonly affecting the scalp.

Scanning electron microscopy (SEM) with a Jeol microscope, JSM ‒ 6610LV at CEME-SUL (microscopy center of the southern region, of the Universidade Federal do Rio Grande) was used to examine a colony of *P. hortae* obtained from the mycolibrary of Instituto de Medicina Tropical de São Paulo, lineage 499, with the aim of describing its ultrastructure.

## Results

**Fig. 2:**
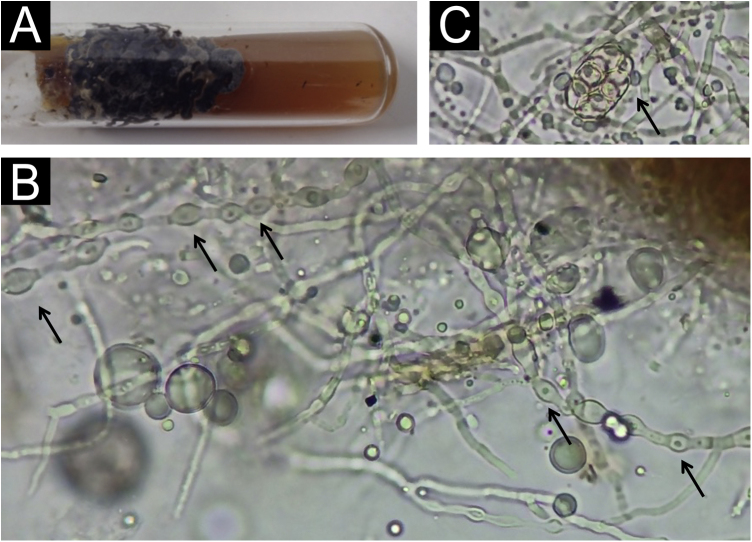
(A) Blackish appearance of the colony. (B) Optical microscopy – dilated hyphae (arrows); in the upper right corner, one can see the brownish area of the colony, which was not dissolved. (C) Optical microscopy ‒ initial ascospore (arrow).

The colony has a typical blackish appearance (Fig. 2A). Examination of the microculture with optical microscopy demonstrates hyphae with typical dilations (Fig. 2B), as shown in Fig. 1B. Ascospores (Fig. 2C) were also observed on optical microscopy, showing a structure similar to drawing 10 in Fig. 1A.

It is noteworthy that, in the potassium hydroxide test, part of the colonies do not dissolve, forming brownish clumps, which are difficult to focus and examine due to their thickness (Fig. 2B).

**Fig. 3:**
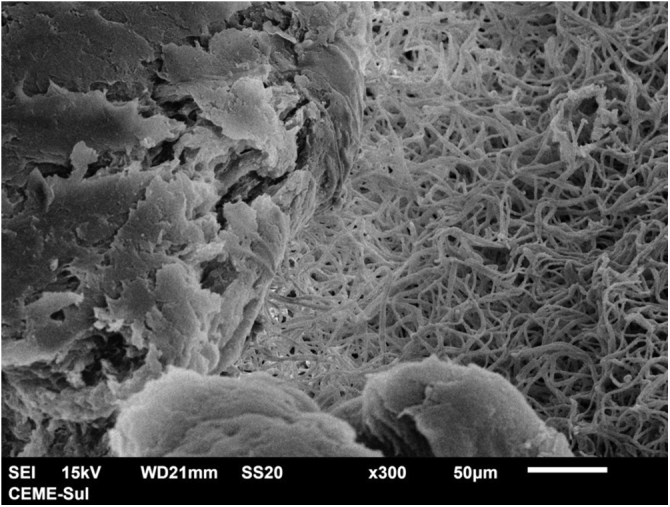
Scanning electron microscopy (SEM) - low magnification viewing the colony with filamentous area on the right and compact area on the left (×300).

With scanning electron microscopy on low magnification, two distinct aspects of the colony can be identified, a compact area and a filamentous one (Fig. 3).

**Fig. 4:**
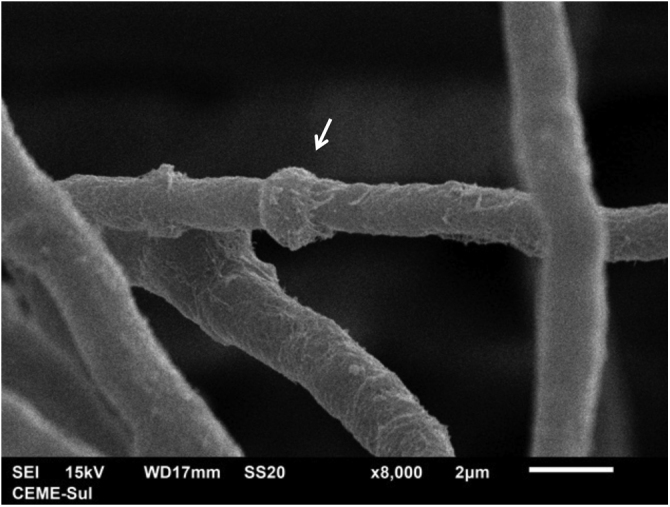
SEM ‒ detail of a dilated hyphae (arrow), similar to those seen under optical microscopy (×8,000).

**Fig. 5:**
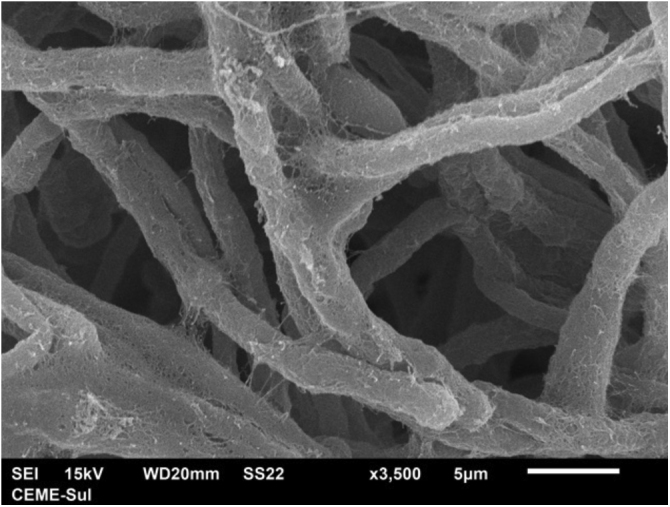
SEM – hyphae covered with microfibrillar extracellular matrix (×3,500).

**Fig. 6:**
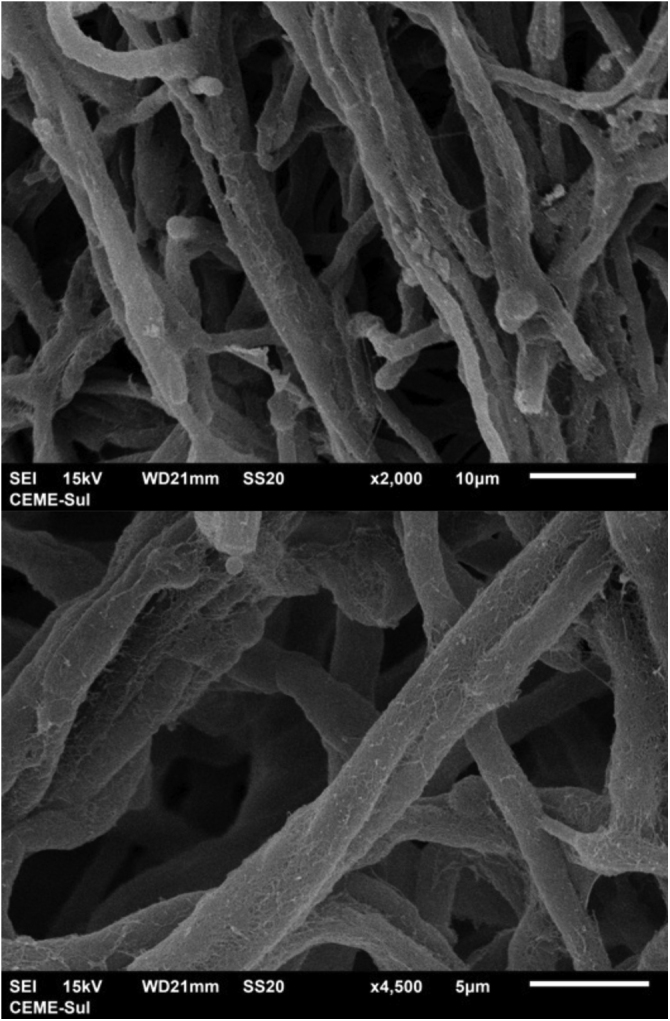
SEM ‒ adhered and parallel hyphae (×2,000 and ×4,500).

**Fig. 7:**
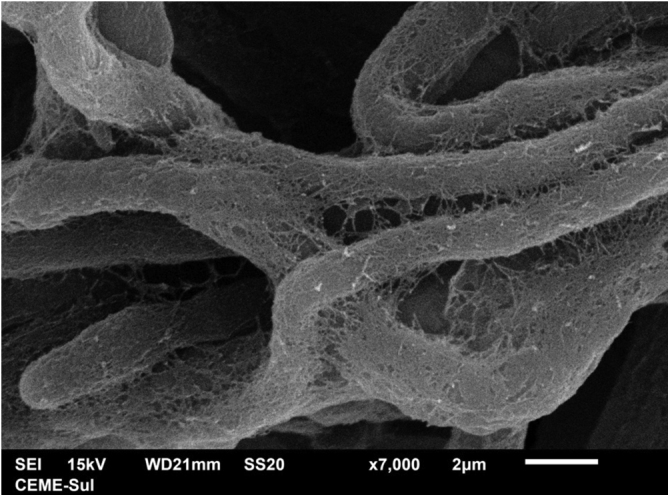
SEM ‒ high magnification, showing in detail hyphae adhered by the reticular matrix (×7.000).

**Fig. 8:**
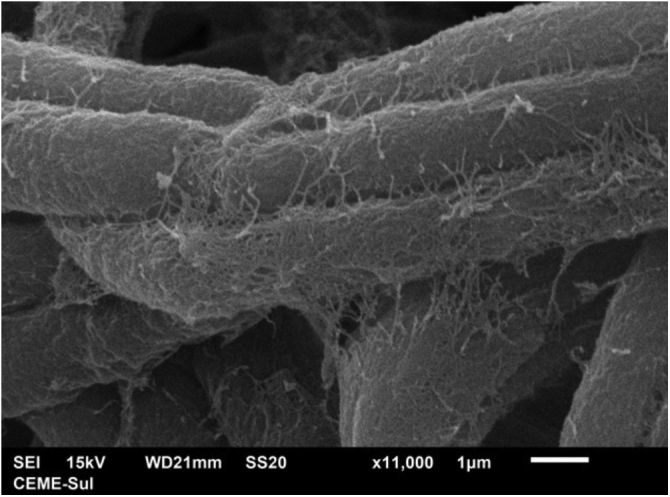
SEM ‒ high magnification with detail of the adhesion and parallel arrangement of hyphae (×11.000).

Examination of the filamentous area demonstrates dilations in the hyphae, as seen under optical microscopy (Fig. 4). Detailed examination of them also shows that they are adhered by a thin reticular substance (Fig. 5). A recurring finding is the adhesion between the fungal filaments in a parallel distribution (Fig. 6). On high magnification, the micro-fibrillar substance adhering the hyphae to each other becomes very evident (Fig. 7, Fig. 8).

**Fig. 9:**
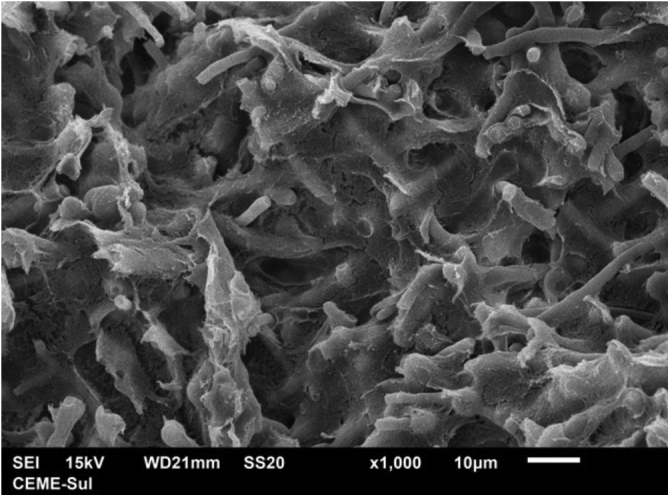
SEM – low magnification in the compact area, showing fungal structures embedded in dense extracellular matrix (×1.000).

**Fig. 10:**
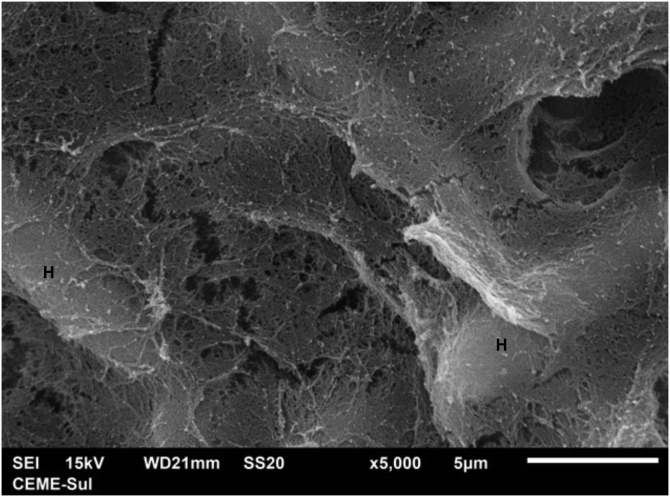
SEM – high magnification in the compact area, depicting hyphae (H) embedded in microfibrillar matrix (×5.000).

Examination of the compact area shows the hyphae embedded in the reticular matrix forming a biofilm (Fig. 9), and the colony looks well adhered. On high magnification, it can be observed that the hyphae are inside this fibrillar matrix (Fig. 10), which has the same appearance as the extracellular matrix that adheres the hyphae to each other in the filamentous area.

## Discussion

No reports were found about the ultrastructural examination of *P. hortae* colonies, only of the disease nodules; in these reports, a cementing extracellular substance^10^, ^11^ is mentioned, as forming the nodules on the hair shafts, together with hyphae and spores.

The present findings demonstrate that the fungal structures produce a substance secreted into the extracellular environment, with a microfibrillar appearance, which adheres the hyphae to each other, and in some areas, perhaps older areas of the colony, causes great compaction, embedding these structures and forming a biofilm. Ascospores were not found, as only the surface of samples is examined using this technique and ascospores are found inside the colony or nodule of the *piedra*.^11^

This fibrillar network is what may provide resistance and configuration to *Piedra* nodules, allowing them to occur in the partly hostile environment of the hair shafts, possibly also acting as a factor for hair adhesion in disease dissemination. Corroborating this formation of resistant structures, upon direct examination with optical microscopy, there is some difficulty in dissolving the colonies, which appear as brownish clumps, as seen in figure 2B.

The term biofilm was first used in the 1970s, despite being an old observation by microbiologists. It describes a polymeric extracellular matrix with embedded etiological agents, having a protective function against ultraviolet radiation, extreme temperatures and pH, salinity, and pressure that are harmful to bacteria, as well as being involved in antibiotic resistance.^12^ They can be formed by polysaccharides, proteins, or fats,^12^ and with the morphological analysis technique used here, it is not possible to establish the composition of the documented matrix.

Fungi can also produce biofilms, which has already been demonstrated in species that cause onychomycosis such as *Trichophyton rubrum* and *T. mentagrophytes*.^13^, ^14^ In a publication, slight adhesion between the hyphae was described in *T. mentagrophytes* colonies using SEM,^15^ a lighter finding than the one reported herein for *P. hortae*.

Regarding the *Piedras*, the formation of biofilms has already been described in several species of Trichosporon,^16^, ^17^ and interspecies variation has been found, allowing them to be classified as weak or strong producers of biofilms. Possibly, strains with low biofilm production do not cause *Piedra alba*.

The ultrastructural findings of the *Piedraia hortae* colony demonstrate that the formation of biofilm by the extracellular matrix secreted by the hyphae may be crucial in the pathogenesis of *Piedra nigra*.

## Financial support

None declared.

## Authors' contributions

Hiram Larangeira de Almeida Jr.: Approval of the final version of the manuscript; design and planning of the study; drafting and editing of the manuscript; collection, analysis and interpretation of data; effective participation in research orientation; intellectual participation in the propaedeutic and/or therapeutic conduct of the studied cases; critical review of the literature; critical review of the manuscript.

Thales de Moura Assis: Approval of the final version of the manuscript; design and planning of the study; drafting and editing of the manuscript; collection, analysis and interpretation of data; intellectual participation in the propaedeutic and/or therapeutic conduct of the studied cases; critical review of the literature; critical review of the manuscript.

Eduardo Camargo Faria: Approval of the final version of the manuscript; design and planning of the study; drafting and editing of the manuscript; collection, analysis and interpretation of data; intellectual participation in the propaedeutic and/or therapeutic conduct of the studied cases; critical review of the literature; critical review of the manuscript.

Luiz Roberto Kramer Costa: Approval of the final version of the manuscript; design and planning of the study, drafting and editing of the manuscript; collection, analysis and interpretation of data; intellectual participation in the propaedeutic and/or therapeutic conduct of the studied cases; critical review of the literature; critical review of the manuscript.

Berenice Marques Ibaldo: Approval of the final version of the manuscript; design and planning of the study; drafting and editing of the manuscript; collection, analysis and interpretation of data; intellectual participation in the propaedeutic and/or therapeutic conduct of the studied cases; critical review of the literature; critical review of the manuscript.

## Conflicts of interest

None declared.
